# Supplementary material for: Electron-donating amine-interlayer induced n-type doping of polymer:nonfullerene blends for efficient narrowband near-infrared photo-detection
Source: Nat Commun. 2022 Sep 3;13:5194. doi: 10.1038/s41467-022-32845-5 (PMC9440933; doi:10.1038/s41467-022-32845-5)
Supplement: Supplementary file 1 — Supplementary Information [file 41467_2022_32845_MOESM1_ESM.pdf]

Supplementary Information

Electron-donating amine-interlayer induced n-type doping of polymer:nonfullerene blends for efficient narrowband near-infrared photo-detection

Quan Liu[1,2]*, Stefan Zeiske[3], Xueshi Jiang[1,2], Derese Desta[1,2], Sigurd Mertens[1,2], Sam Gielen[1,2], Rachith Shanivarasanthe[1,2], Hans-Gerd Boyen[1,2], Ardalan Armin[3] and Koen Vandewal[1,2]*

[1] Hasselt University, Agoralaan 1, 3590, Diepenbeek, Belgium

[2] IMOMEC Division, IMEC, Wetenschapspark 1, 3590, Diepenbeek, Belgium

[3]Department of Physics, Swansea University, Singleton Campus, Swansea, SA2 8PP UK

*Corresponding author E-mail: quan.liu@uhasselt.be, koen.vandewal@uhasselt.be

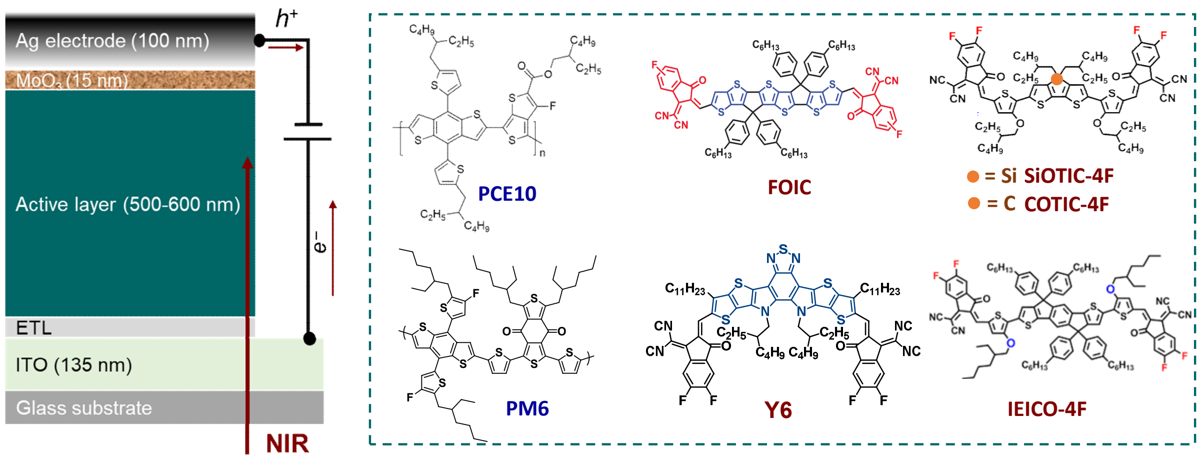

Supplementary Figure 1. Device structure and photoactive materials. Left: inverted thick-junction OPD device architecture. Right: molecular structures of the studied photoactive materials in this work.

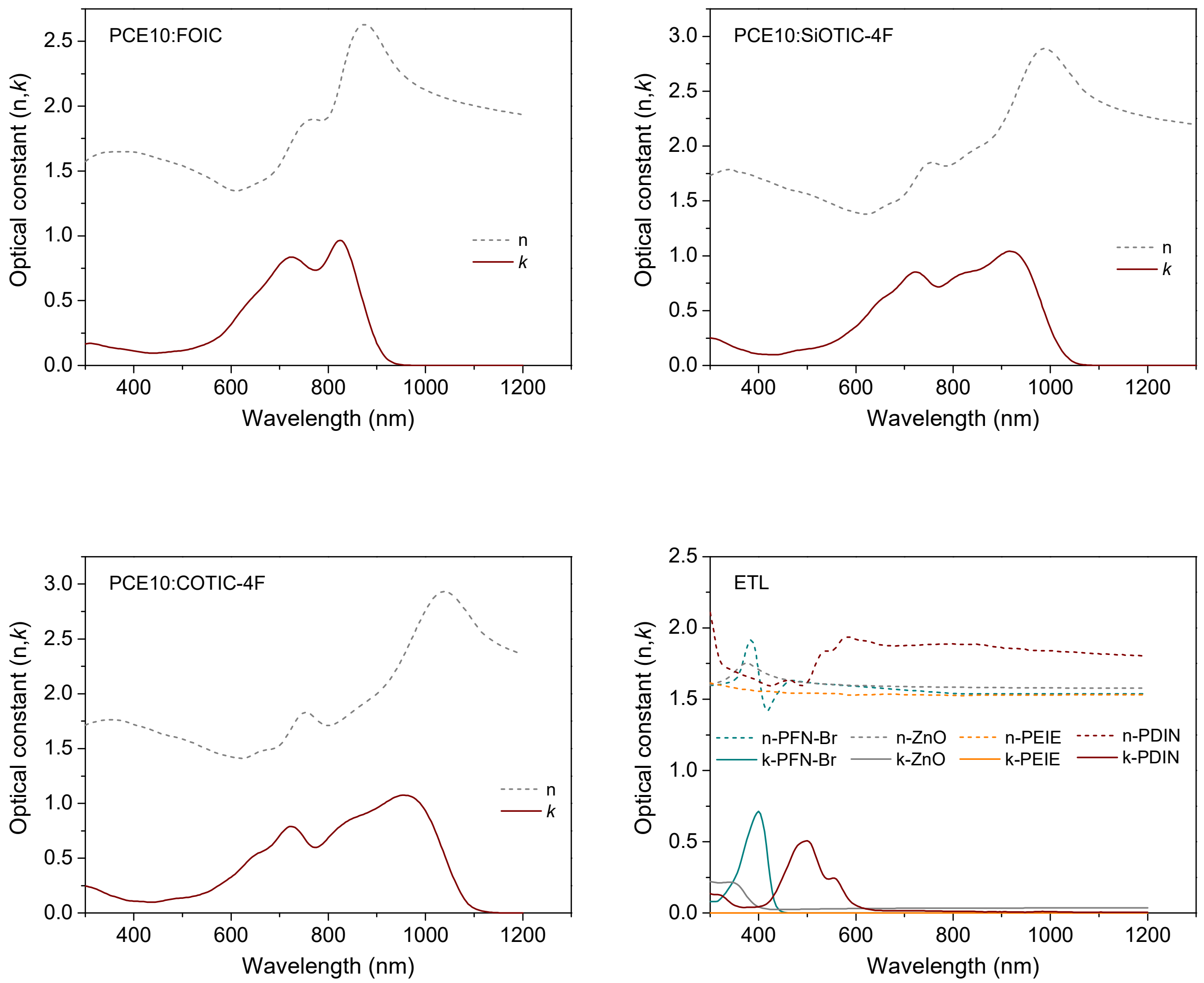

Supplementary Figure 2. Optical constants. Extinction coefficient (*k*) and refractive index (n) of the used photoactive materials and electron transporting layers.

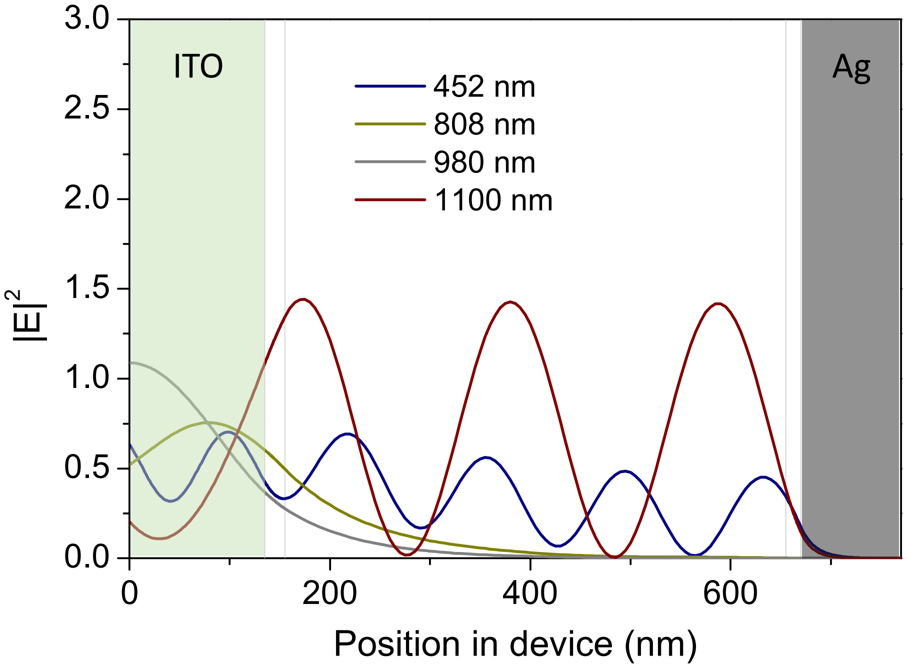

Supplementary Figure 3. **Computed optical field profile for the selected wavelengths**. The field distributions for four wavelengths are simulated in a photodiode configuration of ITO (135 nm)/PDIN (15 nm)/PCE10:COTIC-4F (500 nm)/MoO_3 (15 nm)/Ag (100 nm): 808 nm and 980 nm present the high-α regime where the photocarrier are surface generated near ITO side; 452 nm and 1100 nm present the low-α regime where carrier are volume generated.

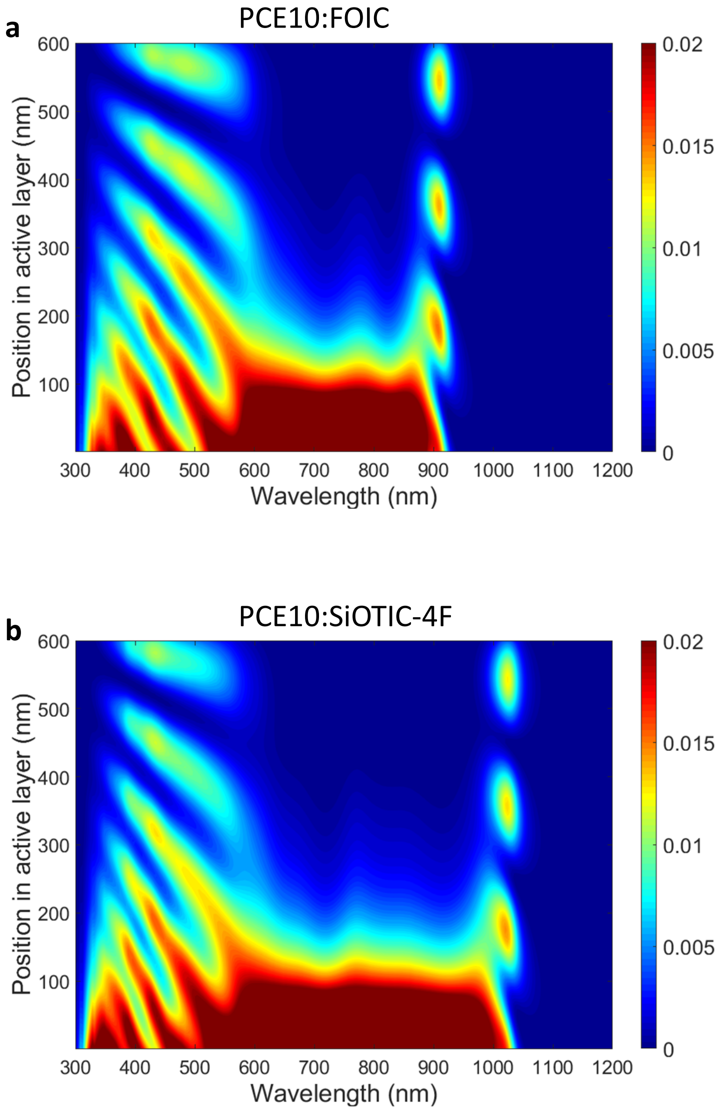

Supplementary Figure 4. Computed spectrally and spatially resolved energy absorption profiles. (**a**) PCE10:FOIC and (**b**) PCE10:SiOTIC-4F. The device architecture used here for simulations is : ITO (135 nm)/PEIE (10 nm)/blend (600 nm)/MoO_3 (15 nm)/Ag (100 nm).

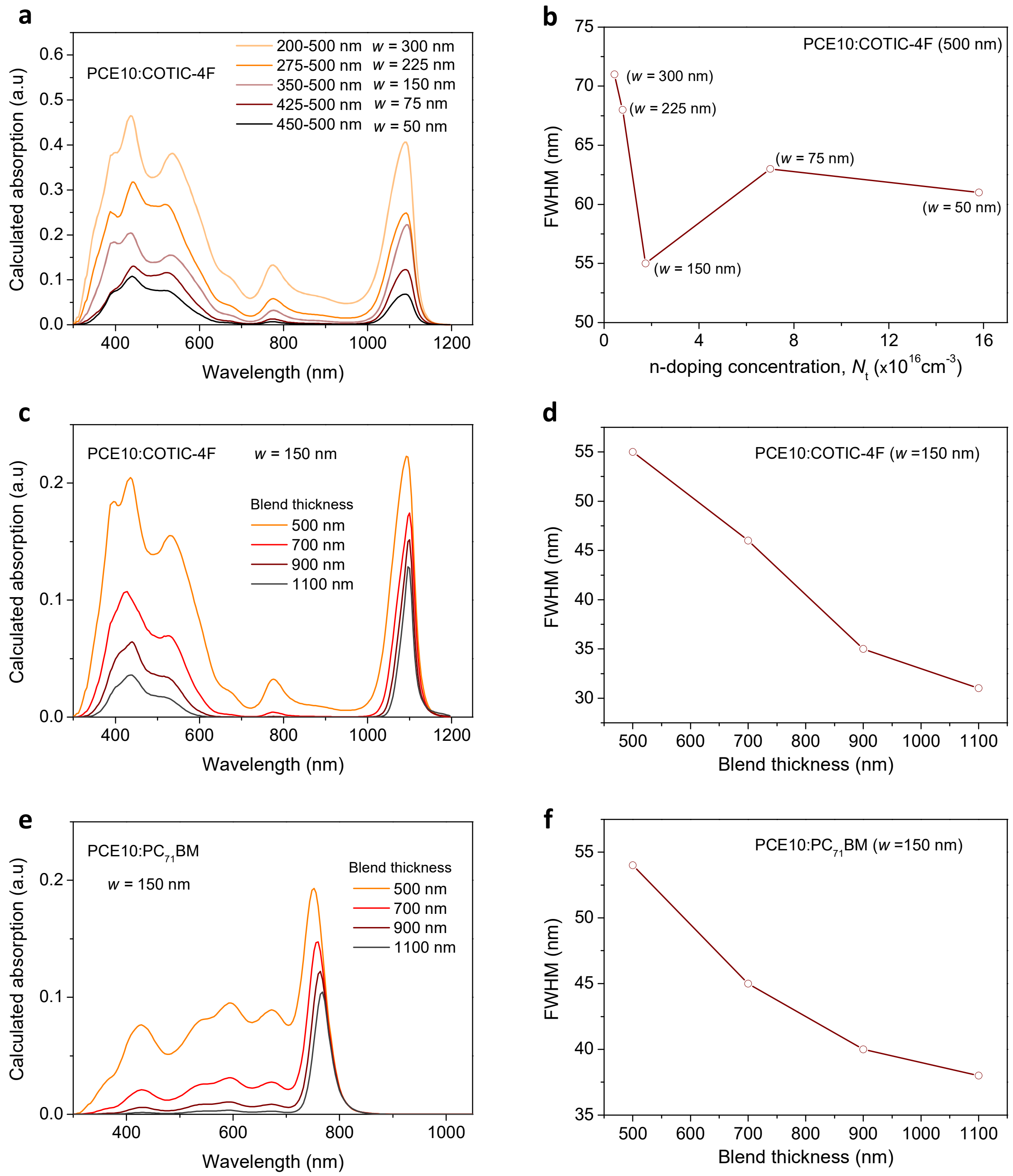

Supplementary Figure 5. **Theoretical study for doping-induced spectral narrowing**. (**a**) TMM computed spatially dependent absorption profiles in the device structure of ITO (135 nm)/PDIN(15 nm)/PCE10:COTIC-4F (500 nm)/MoO3 (15 nm)/Ag (100 nm) with various widths of space charge region near the anode contact. (**b**) FWHM of the calculated narrowband response in the NIR as a function of the n-doping concentration (N_t) or the width of the SCR (w). (**c**) and (**e**) are calculated thickness-dependent absorption spectra of PCE10: COTIC-4F and PCE10:PC_{71}BM OPDs with a fixed SCR width of 150 nm, respectively. (**d**) and (**f**) are the corresponding FWHM plots of the resulting narrowband responses in the NIR region as a function of the blend thickness.

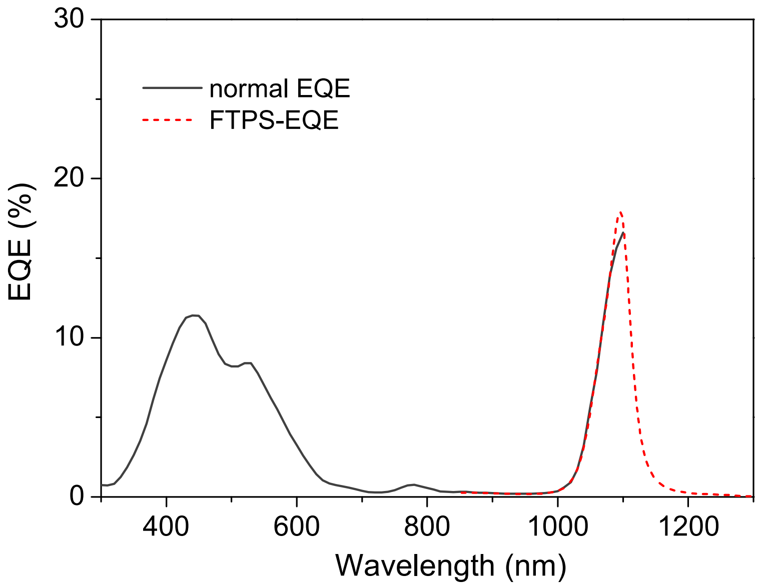

Supplementary Figure 6. **Determination of full EQE spectrum for the PCE10:COTIC-4F device.** By scaling the sensitive FTPS-EQE (red dash line) to the normal EQE (black solid line) spectrum, the longer wavelengths beyond 1050 nm of the EQE are recovered. A very good overlap between 850 nm and 1050 nm is indicated.

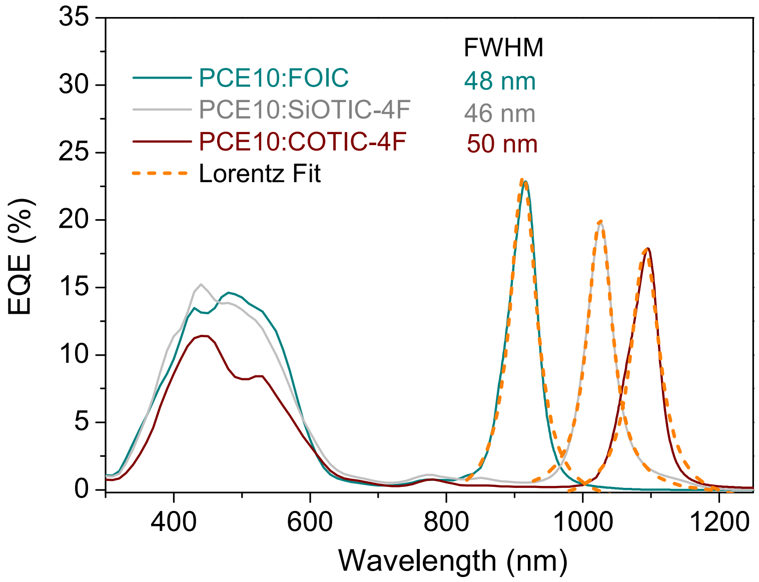

Supplementary Figure 7. Determination of Full-width at half-maximum (FWHM) for narrowband photodetectors. The FWHM of the representative narrowband NIR photodetectors at 918nm,1026 nm and 1096 nm are determined by a Lorentz-fit to their EQE spectra.

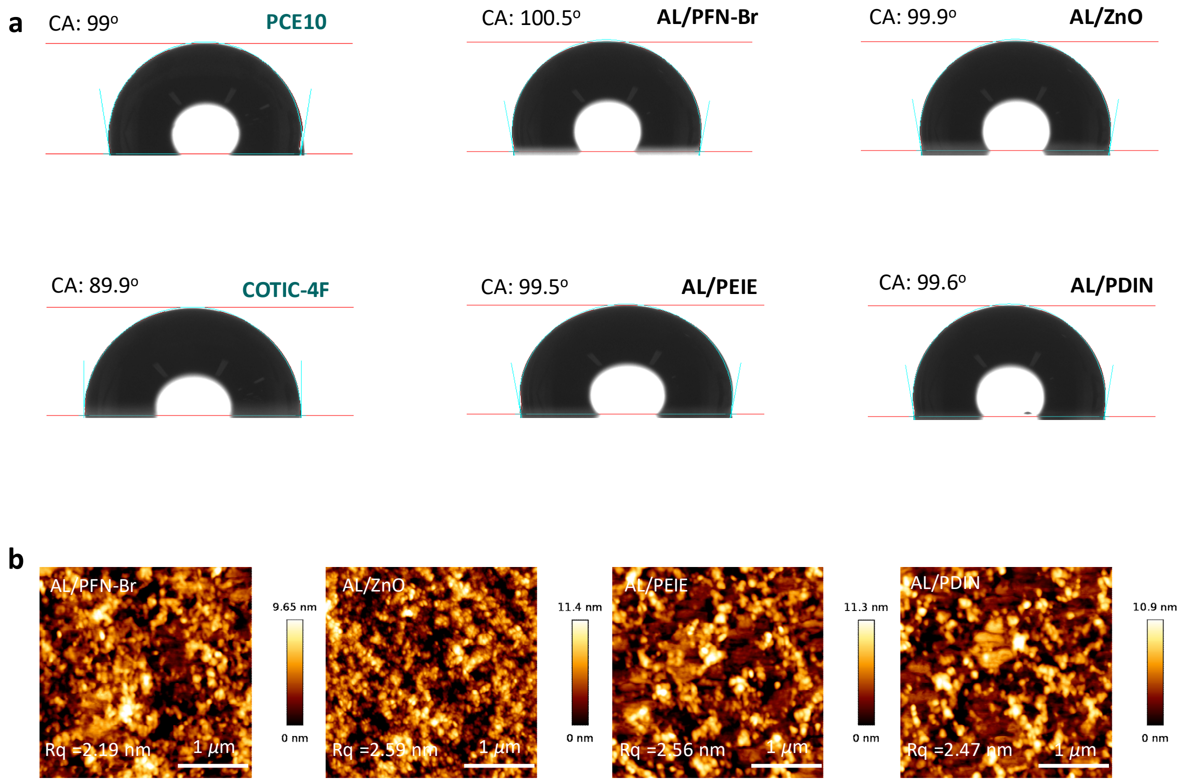

Supplementary Figure 8. **Surface characterization of ETL-dependent PCE10:COTIC-4F films**. (**a**) Static contact angle measurements with water on top of the neat PCE10 polymer, COTIC-4F NFA and ETL-coated PCE10: COTIC-4F blend films (500 nm). All ETL-coated blend films have a very similar contact angle (CA, water) closed to that of neat PCE10 film of 99°, indicating a polymer-rich top surface. (**b**) Topography atomic force microscope (AFM) images. No obvious difference in root mean square (RMS) roughness and significant morphology change is observed amongst the studied samples.

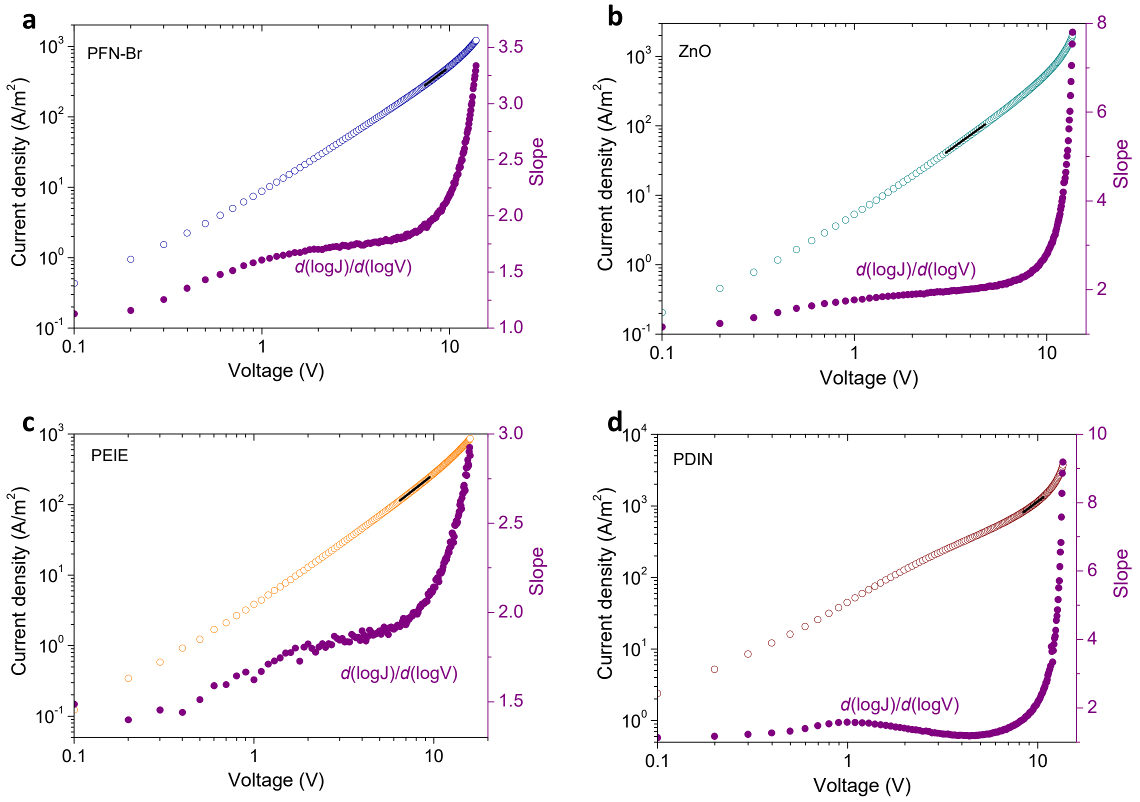

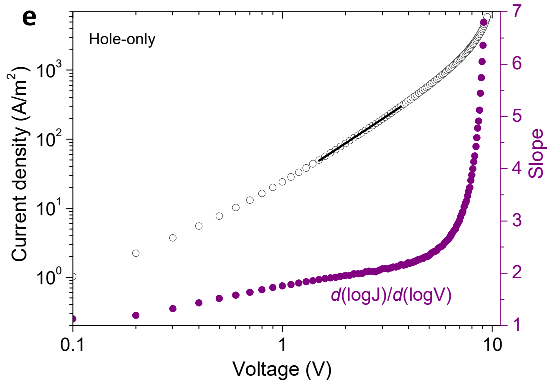

	ETL	mobility (cm^2/Vs)
electron-only	PFN-Br	2.14E-04
	ZnO	1.91E-04
	PEIE	1.13E-04
	PDIN	4.73E-04
hole-only		9.15E-04

Supplementary Figure 9. Mobility measurements. (**a-d**) *J*-V curves of electron-only and (**e**) hole-only PCE10:COTIC-4F devices measured under dark. The mobilities were extracted by fitting the dark *J*–V characteristics of single charge carrier devices using the simple SCLC Mott–Gurney quadratic equation[1], $J_{SCLC}=\frac{9}{8}\varepsilon_0\varepsilon_r\mu\frac{(V_{app}\text{-}V_{bi})^2}{L^3}$, where ε_0 is the permittivity of free space, ε_r is the relative permittivity of the active material (assumed to be 3.5), V_{bi} is the built-in voltage (0.1 V for electron-only device and 0 V for hole-only device, respectively), μ is the mobility, L is the thickness of active layer, V_{app} is the applied voltage corrected by the series resistance induced voltage drop. The solid lines present the best fits in the region where the slope of log(*J*)-log (*V*) plot equals to ≥ 2 but before the onset where the series resistance or tortuosity dominates. The electron- and hole-only device structures are ITO/ETL/PCE10:COTIC-4F (~500 nm)/PFN-Br/Al and ITO/PEDOT:PSS/PCE10:COTIC-4F (~500 nm) /MoO_3/Au, respectively.

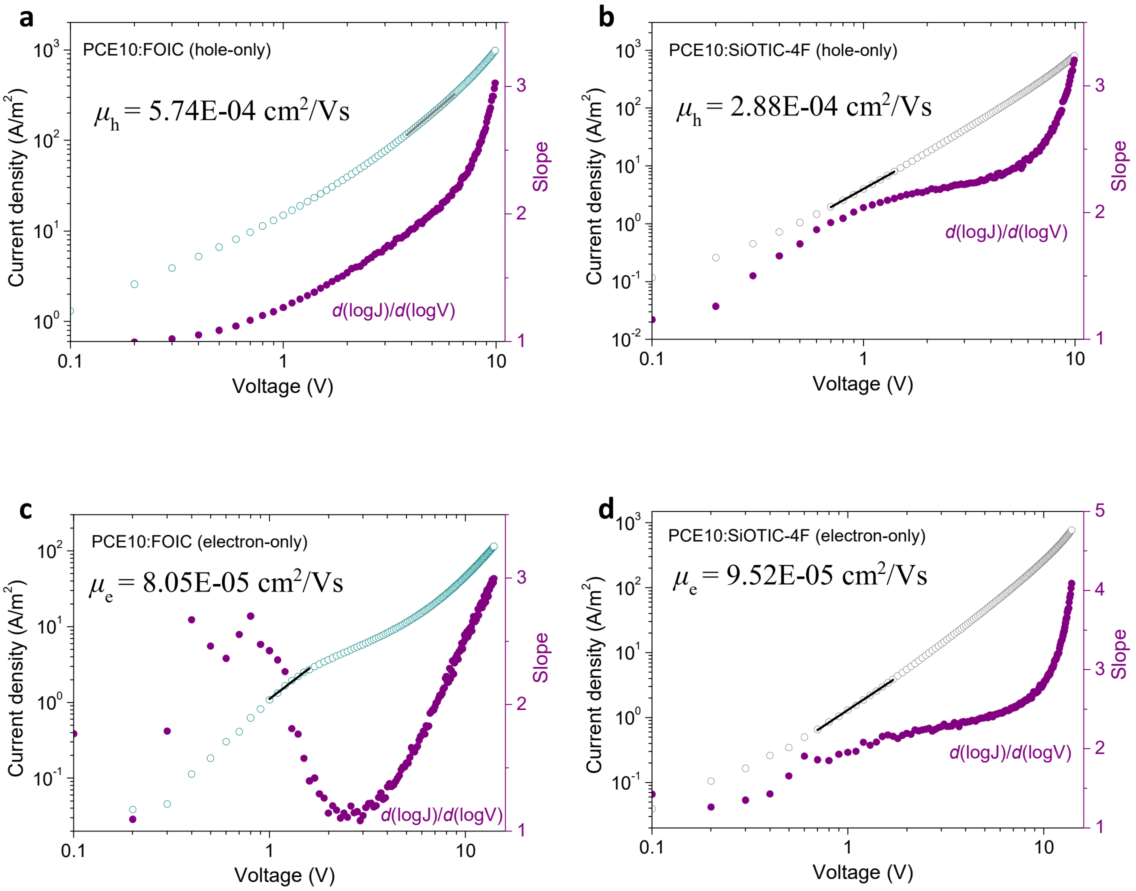

Supplementary Figure 10. Mobility measurements. *J*-V curves of hole-only and electron-only devices measured under dark for PCE10:FOIC (**a**, **c**) and PCE10:SiOTIC-4F (**b**, **d**) respectively. The electron-only device stucture here is ITO/ZnO/Active layer/PFN-Br/Al with a blend thickness of around 600 nm. The extracted mobilities are indicated on each figure.

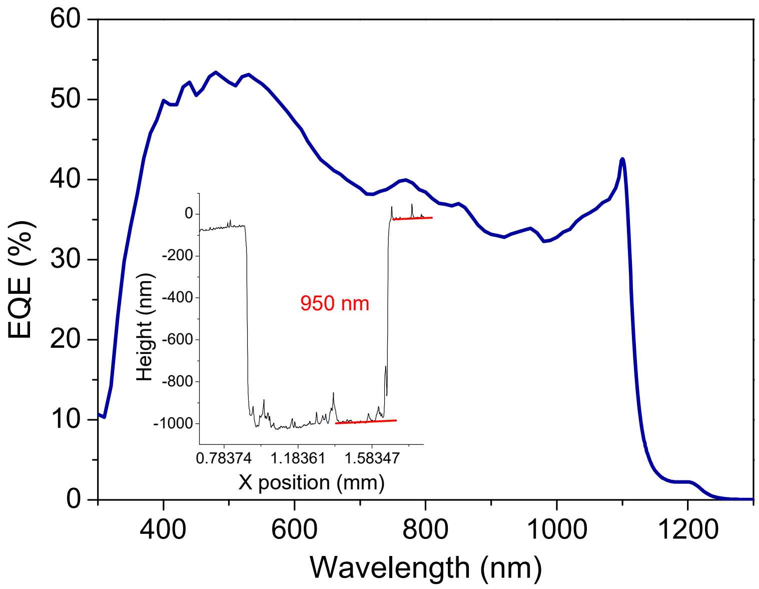

Supplementary Figure 11. Measured EQE spectrum for a ~1 μm-thick OPD. The device structure here is ITO/ZnO (20 nm)/PCE10:COTIC-4F (950 nm)/MoO_3 (15 nm)/Ag (100 nn).

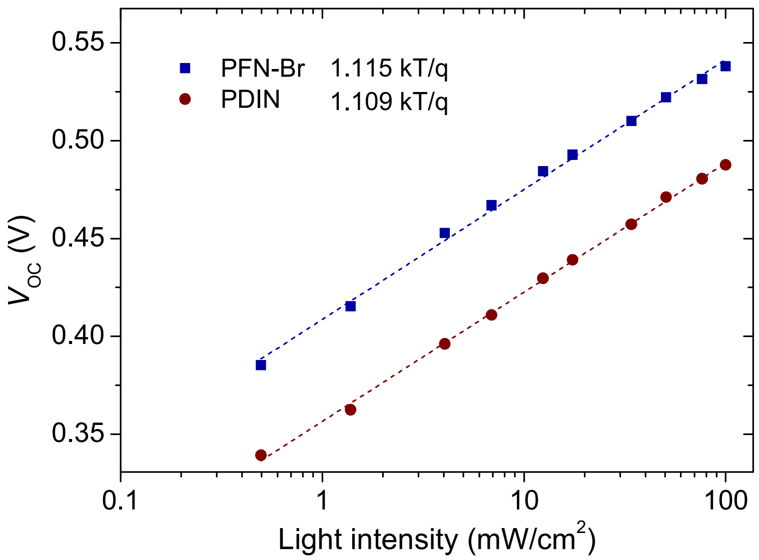

Supplementary Figure 12. light intensity dependent V_{OC}. Semi-log plots of the measured V_{OC} as a function of light intensity for 500 nm-thick PFN-Br and PDIN-PCE10:COTIC-4F inverted devices. The dash lines present the best linear fits.

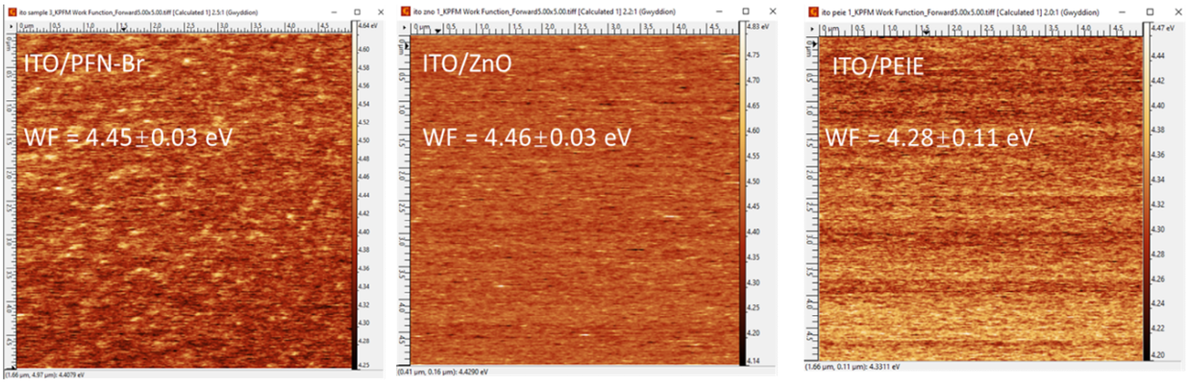

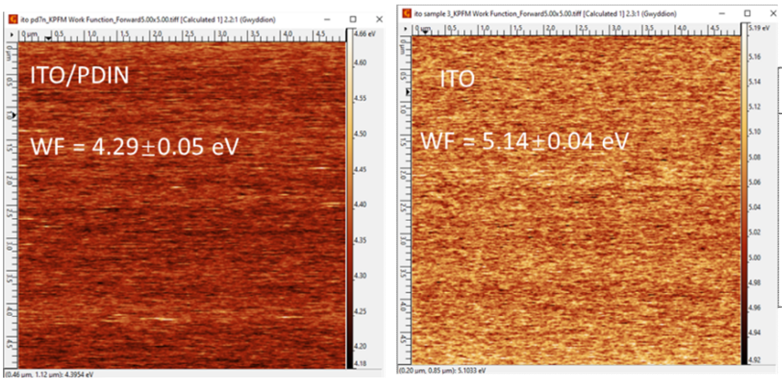

	A	B	C	Average WF (eV)	STD (eV)
ITO	5.185	5.159	5.09	5.14	0.04
ITO/PFN-Br	4.474	4.454	4.41	4.45	0.03
ITO/ZnO	4.43	4.448	4.493	4.46	0.03
ITO/PEIE	4.33	4.382	4.122	4.28	0.11
ITO/PDIN	4.336	4.3	4.219	4.29	0.05

Supplementary Figure 13. Kelvin probe force microscopy (KPFM) measurements. Work function mappings of ITO samples coated with different electron transporting layers (PFN-Br, ZnO, PEIE and PDIN) are recorded by KPFM under dark ambient condition. The average work function and standard deviation for each sample were summarized in the table (bottom right) by measuring three different positions. The measurements were done by frequency modulated KPFM from Park NX10 with a vertical resolution of 0.015 nm and lateral resolution of 0.05 nm. *Pt/Ir* coated tips from ST instruments were used to perform the experiment. Before the measurements, the conductive tips are calibrated and the work function values are calculated from the contact potential difference values obtained from the KPFM and it is given by

$$V_{CPD}=\frac{\varphi_{tip}-\varphi_{sample}}{-e}$$

where V_{CPD} is the contact potential difference φ_{tip} and φ_{sample} are the work function of the tip (~ 5.1 eV) and sample respectively, e is the electronic charge. Note that the work function of the tip we always calibrate before starting any experiment.

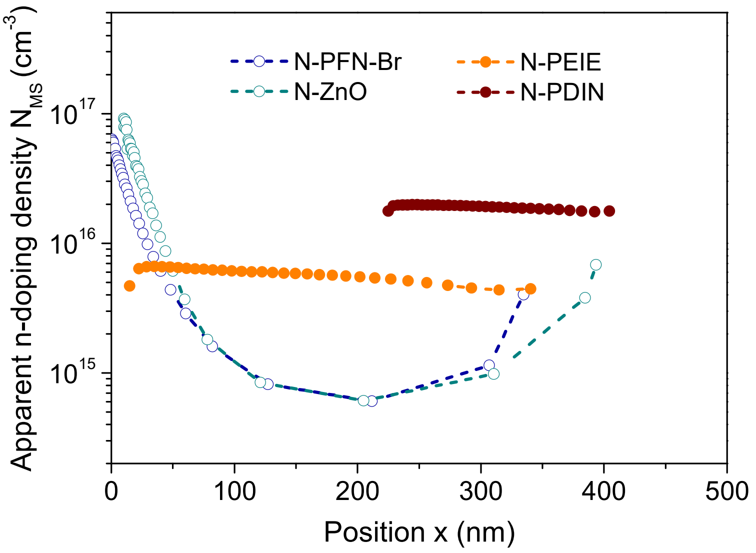

Supplementary Figure 14. Plots of apparent doping concentration. Apparent n-doping density N_{MS} as a function of active layer position x, showing for PCE10: COTIC-4F inverted devices on different ETL substrates. The main junction is at the MoO3/Ag back contact, Here $x = d - \varepsilon A/C$, and d is the active layer thickness (500 nm).

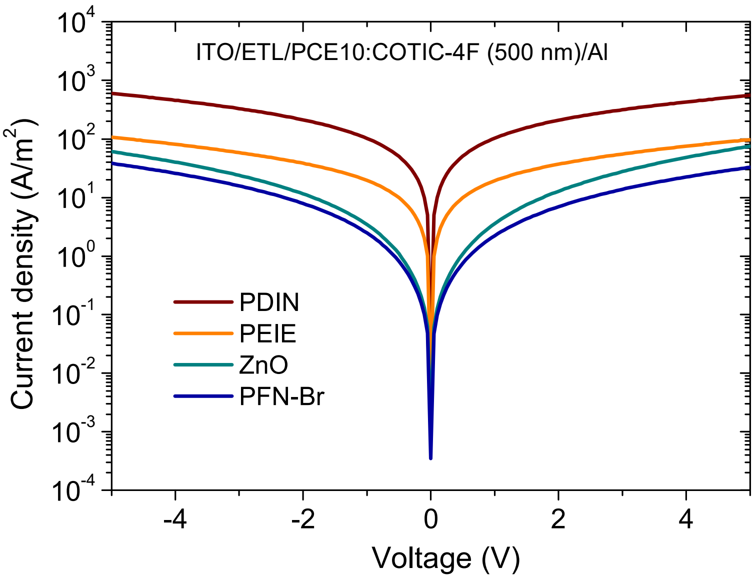

Supplementary Figure 15. **Conductivity characterization.** Semi-logarithmic current density versus voltage characteristics of ETL-dependent electron-only devices measured under dark.

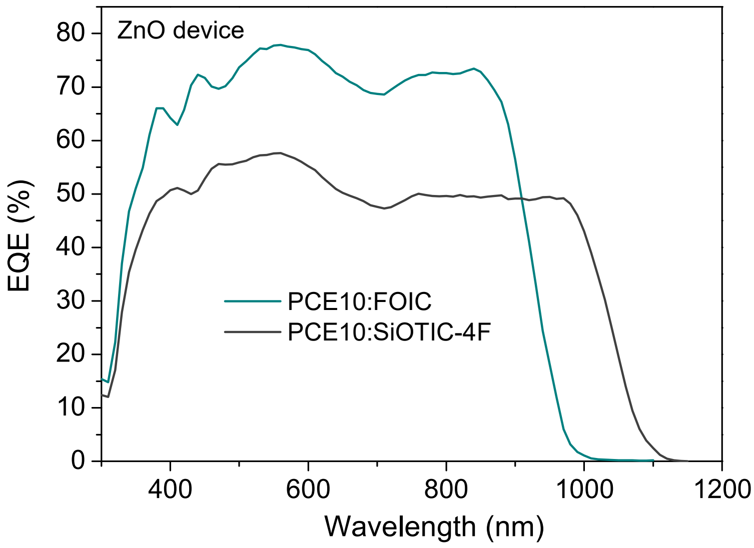

Supplementary Figure 16. **EQE spectra for ZnO devices.** Plots of EQE spectra for both PCE10:FOIC and PCE10:SiOTIC-4F non-doped OPDs showing broadband characteristics. The device structure here is ITO/ZnO (20 nm)/Active layer (~600 nm)/MoO_3 (15 nm)/Ag (100 nn).

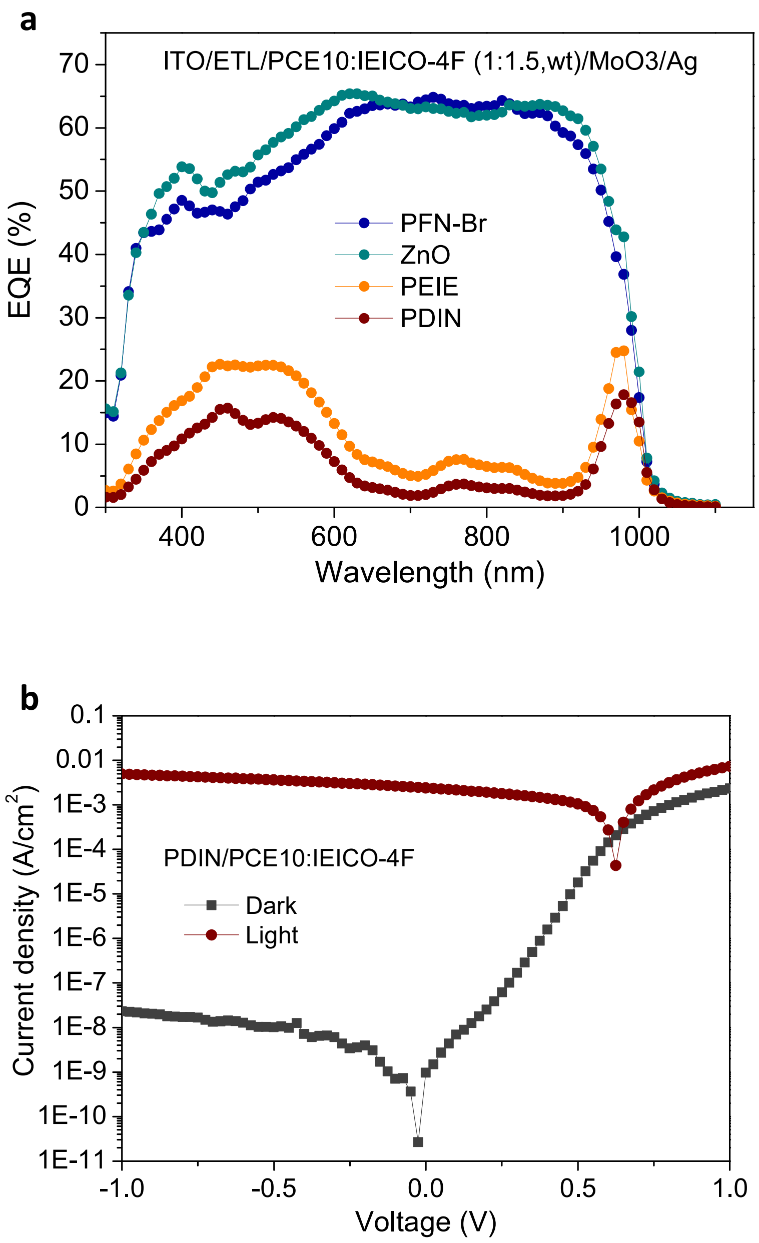

Supplementary Figure 17. Performance of PCE10:IEICO-4F OPD. (**a**) ETL dependent EQE spectra in a 500 nm-thick PCE10:IEICO-4F inverted deivce. (**b**) Light and dark *J*-V curves of PDIN based narrowband NIR-OPD. Note that the blend is prepared by dissolving PCE10 and IEICO-4F with a weight ratio of 1: 1.5 in CB at a total concentration of 40 mg/mL. Spin-coating at 1000 rpm yields around 500 nm-thick active layer.

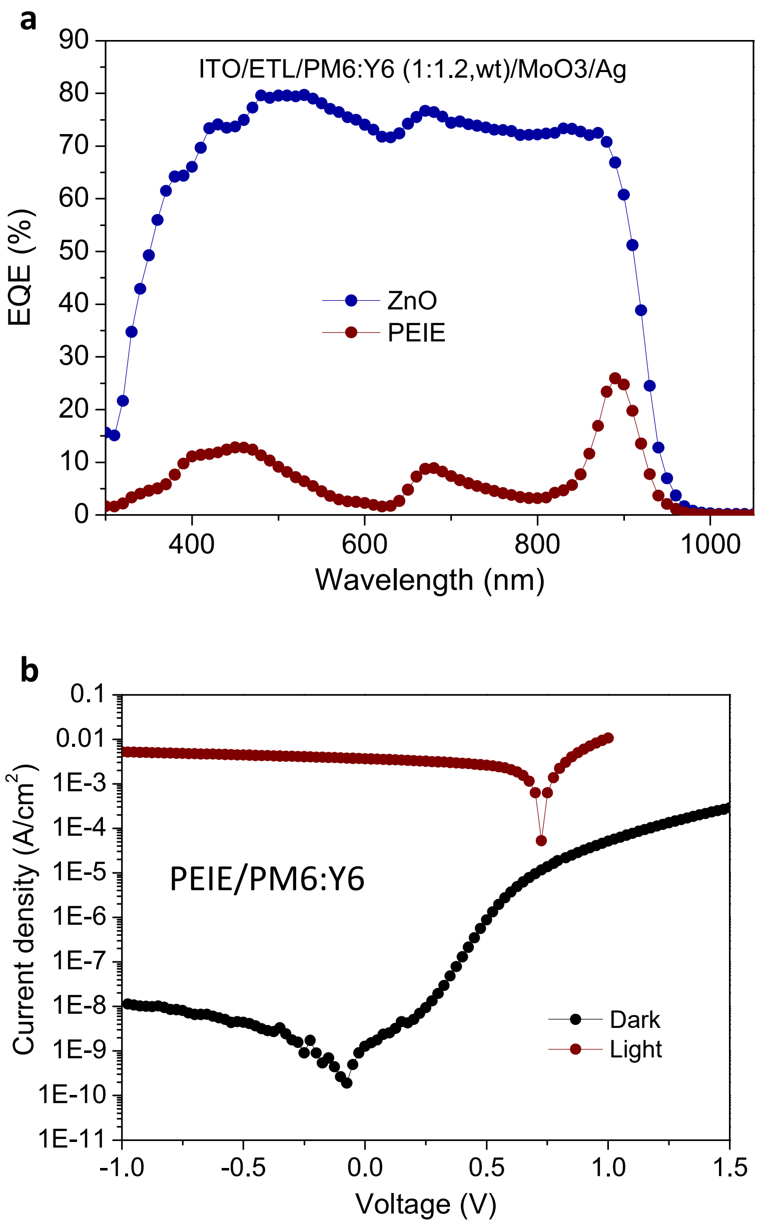

Supplementary Figure 18. **Performance of PM6:Y6 OPD.** (**a**) ETL dependent EQE spectra in a 600 nm-thick PM6:Y6 inverted deivce. (**b**) Light and dark *J*-V curves of PEIE based narrowband NIR-OPD. Note that the blend is prepared by dissolving PM6 and Y6 with a weight ratio of 1: 1.2 in CF at a total concentration of 30 mg/mL. Spin-coating at 1200 rpm yields around 600 nm-thick active layer.

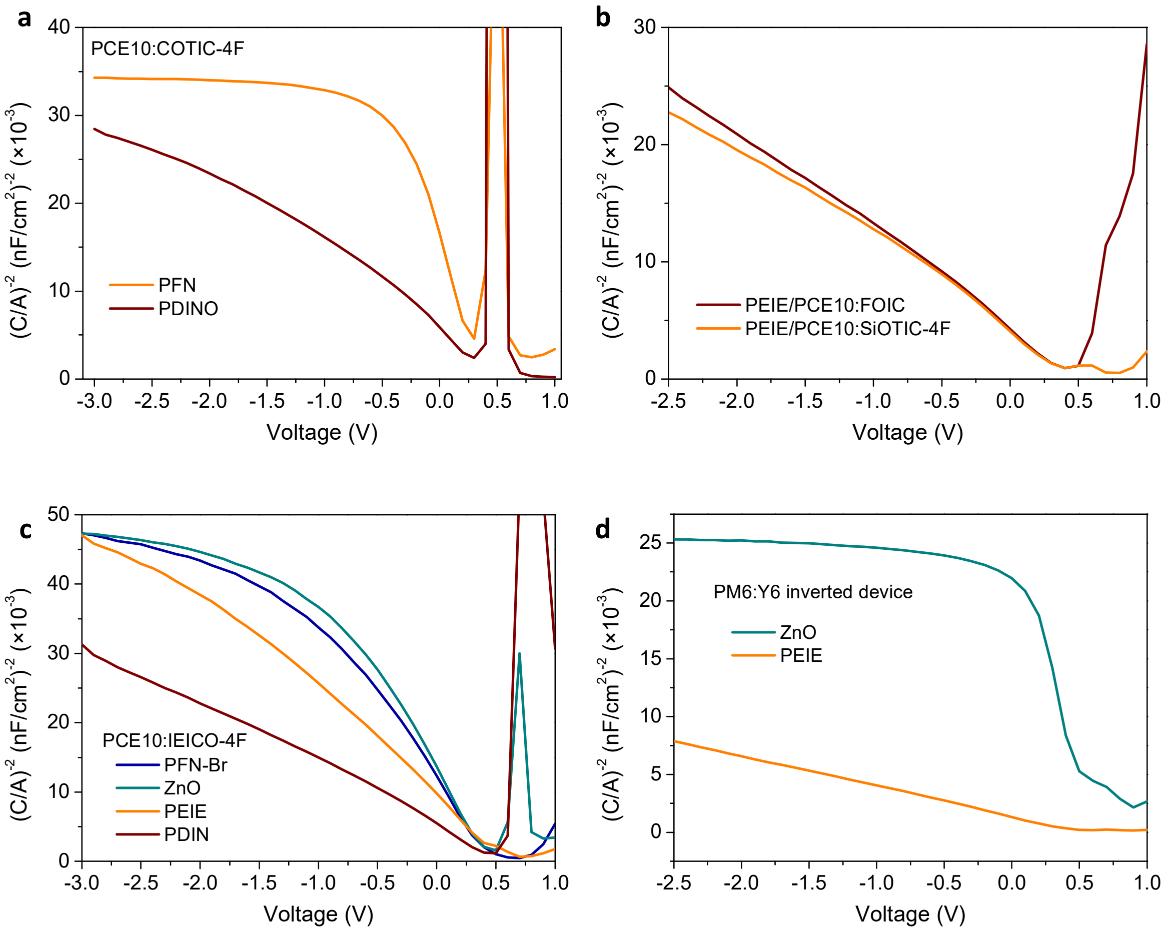

Supplementary Figure 19. **Capacitance-Volatge characteristics**. (**a**) C^{-2}-V plots for PFN and PDINO interlayer based 500 nm-thick PCE10:COTIC-4F devices. (**b**) C^{-2}-V plots for 600 nm-thick PCE10:FOIC and PCE10:SiOTIC-4F devices using a PEIE(H_2O) interlayer. (**c**) C^{-2}-V plots for 500 nm-thick PCE10:IEICO-4F devices using different electron-transporting layers. (**d**) C^{-2}-V plots for 600 nm-thick PM6:Y6 devices with ZnO and PEIE interlayers. The calculated doping density and the width of space charge region are summarized in the below Supplementary Table 1.

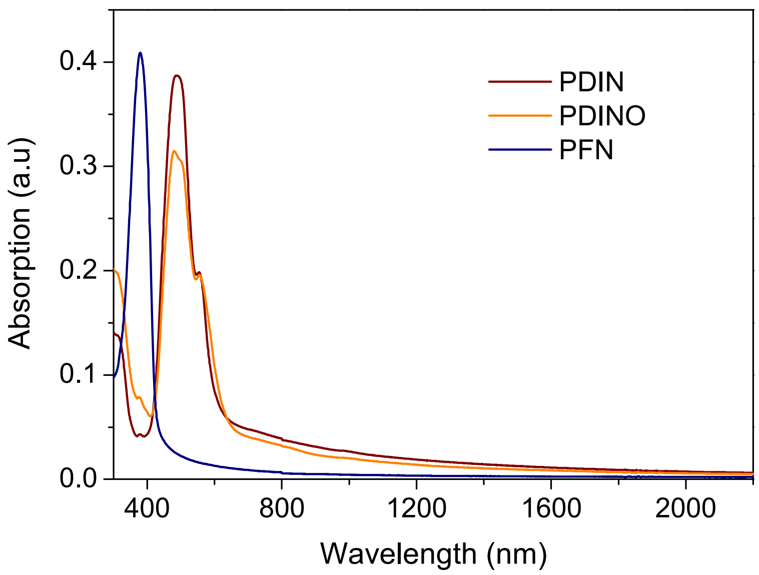

Supplementary Figure 20. Measurements of absorption spectra of electron-transporting layers. UV-VIS-NIR absorption spectra of PFN, PDIN and PDINO thin films.

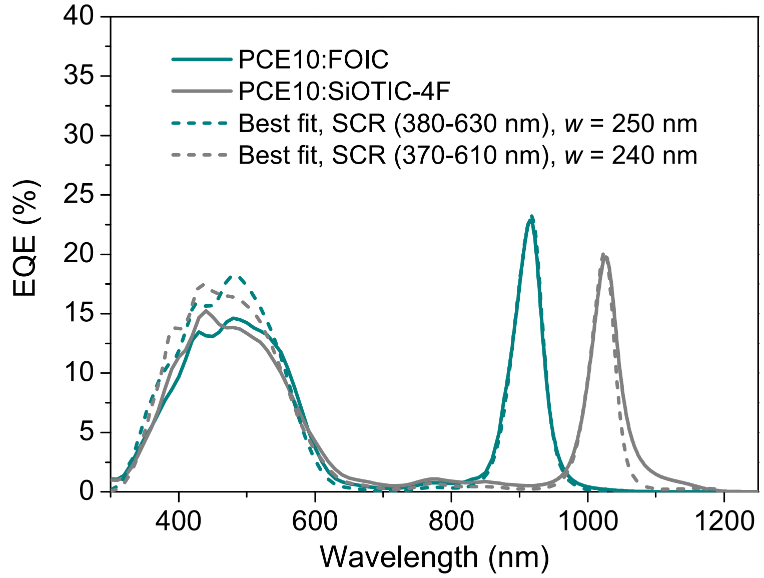

Supplementary Figure 21. **EQE fittings for narrowband NIR-OPDs using a transfer matrix model**. The device architecture here is ITO (135 nm)/PEIE(10 nm)/PCE10:NFA(600 nm)/MoO_3 (15 nm)/Ag (100 nm), and dash lines present the best fits with a constant factor η= 0.8 for PCE10:FOIC and η= 0.75 for PCE10:SiOTIC-4F, repectively. Space charge region (SCR) and corresponding width (*w*) are also indicated.

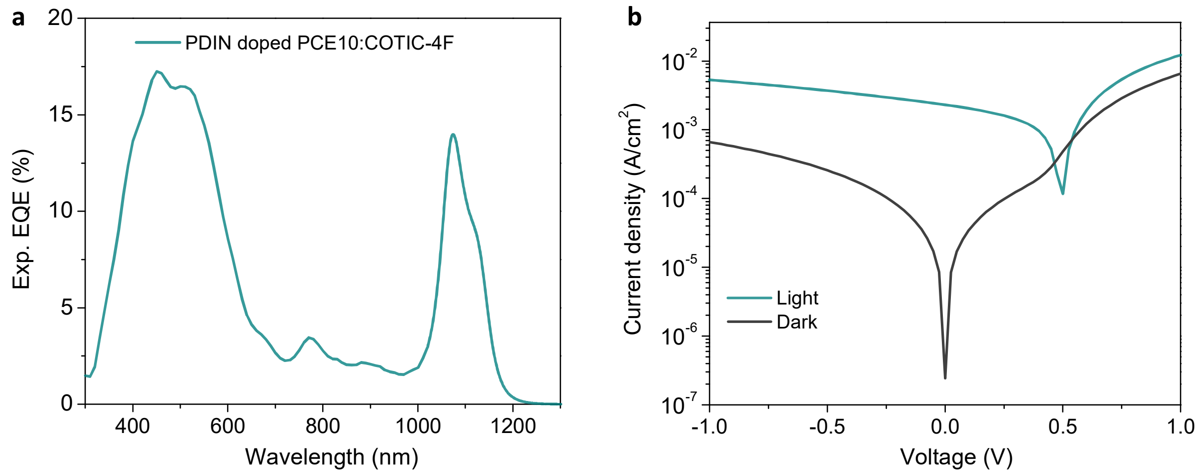

Supplementary Figure 22. **Performance of PDIN-doped 500 nm-thick PCE10:COTIC-4F device**. (**a**) Measured EQE spectrum, and (**b**) light/dark *J*-V curves. Note that the blend solution is prepared by adding PDIN (8mg/mL, prepared in Chloroform+0.5 vol% CH_3COOH) into the PCE10: COTIC-4F (40 mg/mL, CB + 2% vol CN) solution with a volume ratio of 1:20. The increased dark current under reverse bias compared to Fig. 5a in the main text, is mainly ascribed to the morphology change of the solid-state blend film induced PDIN aggregates.

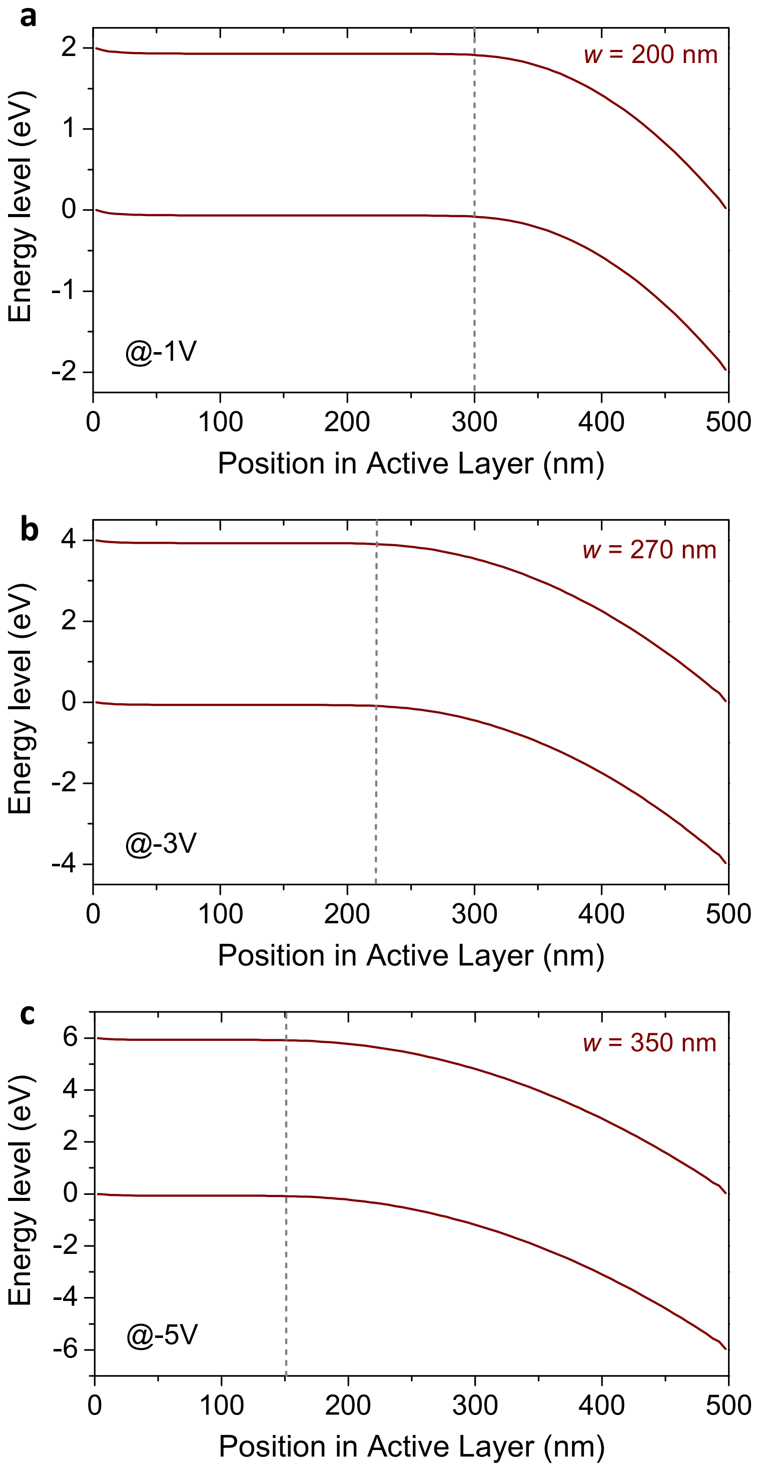

Supplementary Figure 23. **Energy band diagram under negative bias.** Computed energy band diagram for the n-doped device under dark at various reverse bias: (**a**) V = -1 V, (**b**) V = -3 V and (**c**) V = -5 V. The device structure is ITO (135 nm)/PDIN (20 nm)/PCE10:COTIC-4F (500 nm)/MoO_3 (15 nm)/Ag (100 nm) with an n-doping density of N_t =1.88 × 10^{16} cm^{-3}. The width of SCR (w) increases with the applied negative bias.

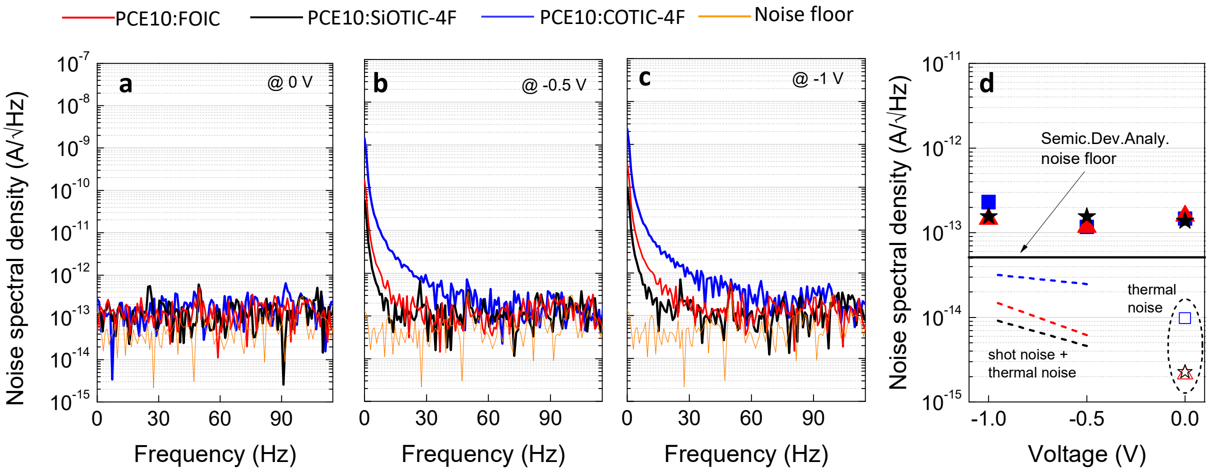

Supplementary Figure 24. Noise measurements. Noise spectral density (NSD) spectra plotted as a function of frequency of PCE10:FOIC (red), PCE10:SiOTIC-4F (black), and PCE10:COTIC-4F (blue) obtained at (**a**) -0 V, (**b**) -0.5 V, and (**c**) -1 V applied bias voltage. Noise currents obtained via FFT-based transient dark current here are consistent with values obtained via direct NSD spectra measurement at higher frequencies. (**d**) Noise currents as obtained *via* NSD (filled symbols) and calculated noise (dashed lines) via $i_{noise}=\sqrt{2qi_d\Delta f+\frac{4k_BT\Delta f}{R_{shunt}}}$ (where q is the elementary charge, k_B is the Boltzmann constant, T is the temperature, i_d is the dark current, R_{shunt} denotes the shunt resistance, and Δf is the electrical bandwidth) plotted as a function of bias voltage. The horizontal solid line marks the noise floor of the Semiconductor Device Analyzer (Keysight B1500A), and open symbols correspond the calculated thermal noise of the three photodetectors at zero applied bias voltage as $i_{noise}=\sqrt{\frac{4k_BT\Delta f}{R_{shunt}}}$. Here, the electrical bandwidth is set to Δf =1 Hz. Note that i_d values here were re-measured at Swansea University, in good agreement with those data shown in Fig. 5a in the main text. The NSD- and J-V-i_d values for each narrowband OPD coincide at high reverse bias voltage due to the suppression of parasitic shunt current effects dominant at small reverse voltages close to 0 V.

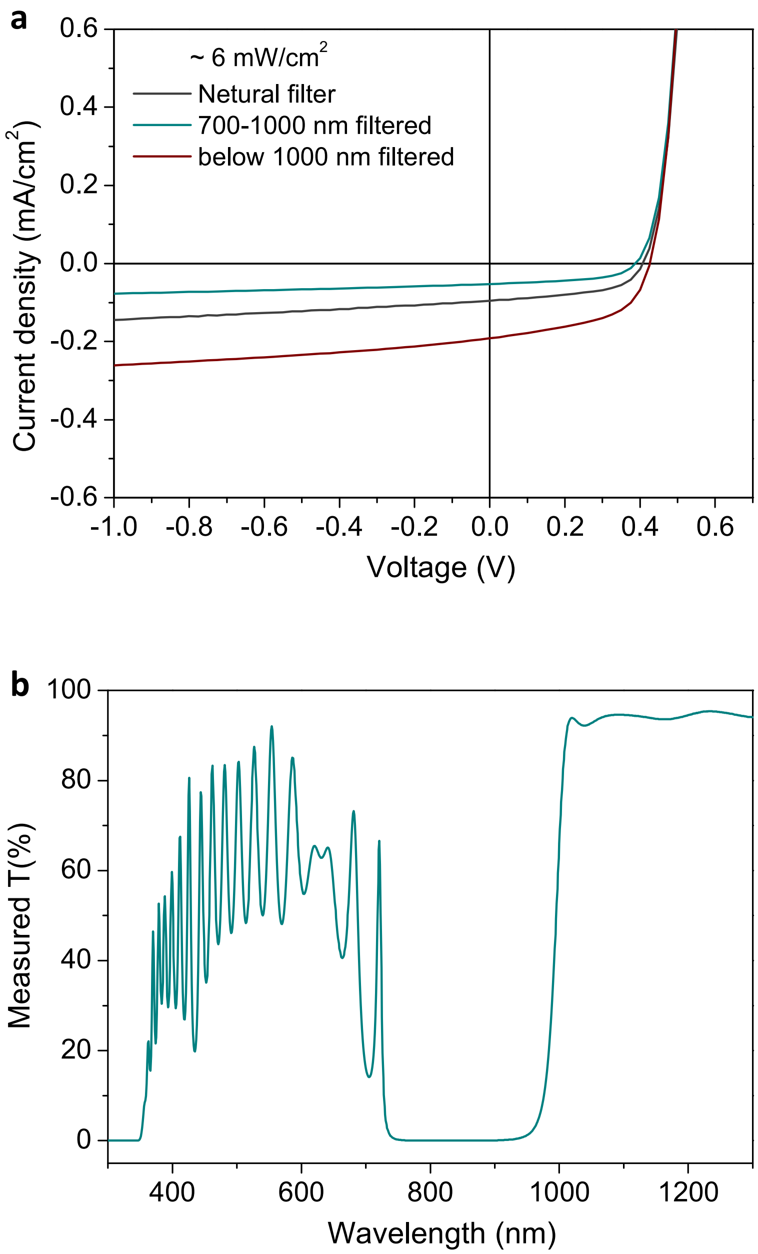

Supplementary Figure 25. **Illumination wavelength dependent current density-voltage characteristics.** (**a**) *J*-V curves of the studied PDIN-based PCE10:COTIC-4F narrowband photodetector, measured under AM 1.5G illumination at a controlled intensity of ~ 6mW/cm^2 with the selected optical filters. Filtering 700-1000 nm was achieved by an optical filter whose transmission spectrum is shown in (**b**). Filtering below 1000 nm was achieved by applying an additional 840 nm cut-off optical filter. All the three *J*-V curves show similar FFs of 52-53%, indicating similar transport properties for the long-lived carriers regardless of the excitation wavelength.

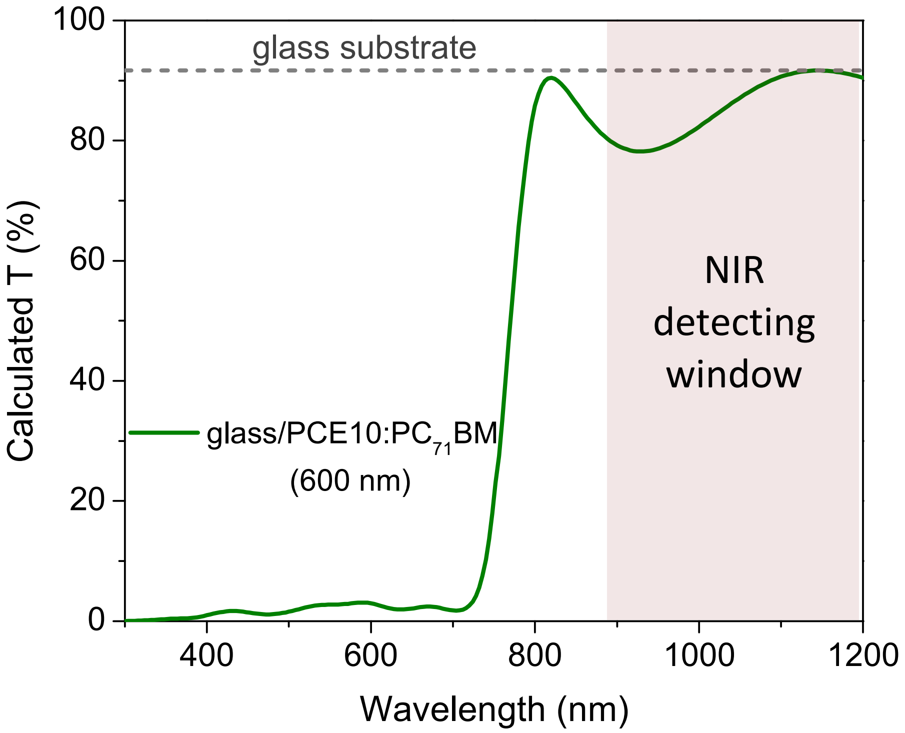

Supplementary Figure 26. **Design for truly visible-blind narrowband NIR-OPDs.** Simulated transmission of glass/PCE10:$PC_{71}BM$ (600 nm), showing a cut-off wavelength of 800 nm. Deposition of such wide-gap layer on the front input side of the near-infrared narrowband photodetectors, truely visible-blind wavelength-selective NIR-detectors are obtained with negligble repsonsivity loss.

Supplementary Note 1. Simulation of energy band diagram

A drift-diffusion model was used to simulate the behavior of carriers in the devices. By solving the below equations, the distributions of free carriers and potential were obtained[2].

$$\nabla \cdot (\varepsilon_r \nabla \phi) = -\frac{q}{\varepsilon_0}\left(p - n + N_D^+ - N_A^-\right) \quad (1)$$

$$J_n = -D_n \nabla n + \mu_n n \nabla \phi \quad (2)$$

$$J_p = -D_p \nabla p + \mu_p p \nabla \phi \quad (3)$$

$$\nabla \cdot J_n = G - R \quad (4)$$

$$\nabla \cdot J_p = G - R \quad (5)$$

Where ϕ is the electrostatic potential, p and n are hole density and electron density. q, ε_0, ε_r, N_D^+ and N_A^- are elementary charge, dielectric constant in vacuum, relative dielectric constant, density of ionized donors and ionized acceptors, respectively. J_n and J_p are electron and hole current densities. G and R are generation rate and recombination rate, respectively.

To take optical effects into account, the profile of exciton generation rate was calculated using transfer matrix method (similar as Fig. 1a in the main text) and then used as the input for drift-diffusion simulations. Work function, mobilities, doping concentration and blend thickness used in simulations were taken from experimental data and were summarized in the below table.

Variable	Unit	Value
work function of anode contact	eV	5.3
work function of cathode contact	eV	4.28
electron mobility (μ_n)	$cm^2V^{-1}s^{-1}$	4.73E-4
hole Mobility (μ_h)	$cm^2V^{-1}s^{-1}$	9.15E-4
effective density of states of conduction band	cm^{-3}	1E+20
effective density of states of valence band	cm^{-3}	1E+20
n-type doping density	cm^{-3}	1.88E+16
p-type doping density	cm^{-3}	0
temperature	K	300
thickness of BHJ blend film	nm	500

Supplementary Table 1. Determination of n-doping concentration and the width of SCR from the measured C-V data.

Device	Slope<br>(d(nF/cm^2)$^{-2}$/dV)	V_{bi}<br>(V)	N_t<br>(n-doping density, cm^{-3})	W<br>(width of SCR, nm)
PFN-Br/PCE10:COTIC-4F	-5.79E+16	1.02	6.99E+14	751
ZnO/PCE10:COTIC-4F	-5.3E+16	1.02	7.63E+14	718
PEIE/PCE10:COTIC-4F	-6.5E+15	1.02	6.22E+15	252
PDIN/PCE10:COTIC-4F	-2.15E+15	1.02	1.88E+16	145
PEIE/PCE10:FOIC	-7.7E+15	1.02	5.25E+15	274
PEIE/PCE10:SiOTIC-4F	-6.7E+15	1.02	6.04E+15	255
PEIE/PM6:Y6	-2.59E+15	1.02	1.56E+16	159
PDIN/PCE10:IEICO-4F	-7.65E+15	1.02	5.29E+15	273

Supplementary Table 2. Performance parameters of narrowband near-infrared photodetectors.

Active material	NIR-OPD wavelength	EQE	R_{shunt}	i_{noise}	Spectral response	D *	FWHM	f_{RC}
	[nm]	[%]	[Ω cm^2]	[A $cm^{-1}Hz^{-1/2}$]	[A W^{-1}]	[Jones]	[nm]	[kHz]
PCE10:FOIC	918	23	1.5E+08	1.06E-14	1.70E-01	1.60E+13	48	1166
PCE10:SiOTIC-4F	1026	20	9.0E+07	1.35E-14	1.65E-01	1.23E+13	46	1205
PCE10:COTIC-4F	1096	18	2.8E+07	2.42E-14	1.59E-01	6.59E+12	50	624
PCE10:IEICO-4F	980	18	4.0E+07	2.03E-14	1.42E-01	7.02E+12	49	
PM6:Y6	892	27	2.0E+08	9.14E-15	1.94E-01	2.13E+13	50	

i_{noise} here is calculated from the equation of $i_{noise}=\sqrt{2qi_d+\frac{4k_BT}{R_{sh}}}$, where i_d is the dark current density, R_{sh} is the shunt resistance derived from the inverse slope of the *J*-V dark curves, q is the elementary charge, k_B is Boltzmann constant, and T is the absolute temperature. We calculated D* for a short-circuit condition, $2qi_d = 0$. *RC* time constant limited cutoff frequency (f_{RC}) is calculated via $f_{RC}=\frac{1}{2\pi\cdot R\cdot C}$, here reisistance R = 150 Ω estimated by summing of the series resistance of device and the load resistance of the oscilloscope; C is the measured capacitance at V = 0 V shown in Fig. 2c (0.91 nF, 0.88 nF and 1.7nF are for PCE10:FOIC, PCE10:SiOTIC-4F and PCE10:COTIC-4F narrowband NIR-OPDs at 1 kHz, respectively).

Supplementary Table 3. Overview of reported narrowband NIR-OPDs to date with λ_{det} ranging from 900 to 1400 nm.

No.	Active material	Narrowband NIR-OPD λ_{det} (nm)	EQE (%)	FWHM (nm)	Bias (V)	D* (Jones)	Ref.
1	SQ-H:PC61BM	1040	12.3	85	0	4.0E+10	3
	SQ-H:PC61BM	1040	20.5	91	-1	1.0E+10	
2	PBTTT:PC61BM (cavity)	915	21	24	0	4.7E+12	4
		960	18	22	0	8.1E+12	
3	PCDTPTSe:PC71BM (cavity)	920	9.7	55	0	1.3E+11	5
		1000	7.5	85	0	1.1E+11	
		1130	6.5	44	0	1.1E+11	
		1190	6.8	41	0	1.2E+11	
		1250	4.2	44	0	7.8E+10	
		1295	3.5	33	0	6.9E+10	
		1390	1.4	30	0	3.0E+10	
4	DPP-DTT:PC71BM	930	8	70	-1	4.7E+12	6
5	DT-PDPP2T-TT/Y6	910	--	43	-0.1	7.0E+12	7
	DT-PDPP2T-TT/IEICO-4F	940	--	66	-0.1	1.6E+13	
6	PBTTT-OR-R:PC61BM (cavity)	990	11	35	0	2.3E+11	8
		1120	7.2	35	0	1.8E+11	
		1240	2	30	0	5.6E+10	
		1340	0.6	32	0	1.7E+10	
7	D8:C60 (cavity)	908	0.4	42	0	2.0E+10	9
		1032	0.4	45	0	2.0E+10	
		1150	0.2	50	0	2.0E+10	
		1244	0.15	59	0	2.0E+10	
		1370	0.1	40	0	2.0E+10	
8	ZnPC:C60 (cavity)	910	21	36	0	3.9E+11	10
		950	18	36	0	3.5E+11	
		995	7	< 40	0	1.4E+11	
		1035	5	< 40	0	1.1E+11	
9	J980 dye	980	3.2	50	-0.5	1.70E+08	11
10	D6:C60 (cavity)	1210	0.24	60	0	1.80E+09	12
11	PDTPQx:PC61BM (cavity)	1016	2.5	44	0	2.20E+10	13
		1140	1	45	0	1.06E+10	
12	PCE10:FOIC	918	23	48	0	1.60E+13	**This work**
	PCE10:SiOTIC-4F	1026	20	46	0	1.23E+13	
	PCE10:COTIC-4F	1096	18	50	0	6.59E+12	
	PCE10:IEICO-4F	980	18	49	0	7.02E+12	
	PM6:Y6	892	27	50	0	2.13E+13	

Supplementary References

[1] Felekidis, N., Melianas, A. & Kemerink, M. Automated open-source software for charge transport analysis in single-carrier organic semiconductor diodes. *Org. Electron.* **61**, 318–328 (2018).

[2] Trukhanov, V. A., Bruevich, V. V. & Paraschuk, D. Y. Effect of doping on performance of organic solar cells. *Phys. Rev. B* **84**, 205318 (2011).

[3] Kim, J. H. et al. An Efficient Narrowband Near-Infrared at 1040 nm Organic Photodetector Realized by Intermolecular Charge Transfer Mediated Coupling Based on a Squaraine Dye. *Adv. Mater*. **33**, 2100582 (2021).

[4] Tang, Z. *et al.* Polymer:Fullerene Bimolecular Crystals for Near-Infrared Spectroscopic Photodetectors. *Adv. Mater.* **29**, 1702184 (2017).

[5] Yang, J. *et al.* Cavity-Enhanced Near-Infrared Organic Photodetectors Based on a Conjugated Polymer Containing [1,2,5]Selenadiazolo[3,4- c]Pyridine. *Chem. Mater.* **33**, 5147–5155 (2021).

[6] Armin, A., Jansen-van Vuuren, R. D., Kopidakis, N., Burn, P. L. & Meredith, P. Narrowband light detection via internal quantum efficiency manipulation of organic photodiodes. *Nat. Commun.* **6**, 6343 (2015).

[7] Xie, B. et al. Self-filtering narrowband high performance organic photodetectors enabled by manipulating localized Frenkel exciton dissociation. *Nat. Commun*. **11**, 2871 (2020).

[8] Vanderspikken, J. *et al.* Tuning Electronic and Morphological Properties for High-Performance Wavelength-Selective Organic Near-Infrared Cavity Photodetectors. *Adv. Funct. Mater.* **32**, 2108146 (2022).

[9] Kaiser, C. et al. Manipulating the Charge Transfer Absorption for Narrowband Light Detection in the Near-Infrared. *Chem. Mater*. **31**, 9325–9330 (2019).

[10] Siegmund, B. et al. Organic narrowband near-infrared photodetectors based on intermolecular charge-transfer absorption. *Nat. Commun*. **8**, 15421 (2017).

[11] Anantharaman, S. B. *et al.* Exploiting supramolecular assemblies for filterless ultra-narrowband organic photodetectors with inkjet fabrication capability. *J. Mater. Chem. C* **7**, 14639–14650 (2019).

[12] Wang, Y. et al. Stacked Dual-Wavelength Near-Infrared Organic Photodetectors. *Adv. Opt. Mater*. **9**, 2001784 (2021).

[13] Vandermeeren, T. et al. A PDTPQx:PC61BM blend with pronounced charge-transfer absorption for organic resonant cavity photodetectors – direct arylation polymerization vs. Stille polycondensation. *Dye. Pigment.* **200**, 110130 (2022).
